# When Medicine Meets Engineering—Paradigm Shifts in Diagnostics and Therapeutics

**DOI:** 10.3390/diagnostics3010126

**Published:** 2013-02-27

**Authors:** Hann Wang, Aleidy Silva, Chih-Ming Ho

**Affiliations:** Department of Mechanical and Aerospace Engineering, University of California, Los Angeles, Los Angeles, CA 90095, USA

**Keywords:** microfluidic system, MEMS transducers, feedback system control (FSC), combinatorial drugs, network medicine

## Abstract

During the last two decades, the manufacturing techniques of microfluidics-based devices have been phenomenally advanced, offering unlimited potential for bio-medical technologies. However, the direct applications of these technologies toward diagnostics and therapeutics are still far from maturity. The present challenges lay at the interfaces between the engineering systems and the biocomplex systems. A precisely designed engineering system with narrow dynamic range is hard to seamlessly integrate with the adaptive biological system in order to achieve the design goals. These differences remain as the roadblock between two fundamentally non-compatible systems. This paper will not extensively review the existing microfluidic sensors and actuators; rather, we will discuss the sources of the gaps for integration. We will also introduce system interface technologies for bridging the differences to lead toward paradigm shifts in diagnostics and therapeutics.

## List of Abbreviations

cDNA: Complementary DNA
CE: Capillary Electrophoresis
DMF: Digital Microfluidics
DTPAs: DNA to Protein Arrays
ERK1/2: Extracellular Signal-Regulated Protein Kinases 1 and 2
ESI: Electrospray Ionization
EWOD: Electrowetting on Dielectrics
FCS: Fluorescent Cross-correlation Spectroscopy
FDA: US Food and Drug Administration
FRET: Fluorescent Resonance Energy Transfer
FSC: Feedback Systems Control
GAPDH: Glyceraldehyde 3-phosphate Dehydrogenase
GC: Gas Chromatography
HIV: Human Immunodeficiency Virus
HMG-CoA: 3-hydroxy-3-methylglutaryl-coenzyme A
HSV-1: Herpes Virus Simplex 1
ITT: *In Vitro* Transcription/Translation
LC: Liquid Chromatography
LOC: Lab-on-a-Chip
LOD: Limit of Detection
MALDI: Matrix-assisted Laser Desorption/Ionization
MEMS: Micro-Electro-Mechanical Systems
MITOMI: Mechanically Induced Trapping of Molecular Interactions
mRNA: Messenger RNA
MS: Mass Spectrometry
PBMCs: Peripheral Blood Mononuclear Cells
PCR: Polymerase Chain Reaction
POC: Point-of-Care
PTMs: Post Translational Modification
siRNA: Small Interference RNA
SNP: Single Nucleotide Polymorphism
SPR: Surface Plasmon Resonance
ssDNA: Single-Stranded DNA
STM: Scanning Tunneling Microscope
TGS: Third Generation Sequencing

## 1. Introduction

### 1.1. Engineering System vs. Bio-Complex System

A universal goal of technological development, including micro/nano technologies, is the advancement of human well-being. Micro-Electro-Mechanical-Systems (MEMS) technology enables us to design and fabricate transducers matching the length scale of a biological cell. Furthermore, the development of nano technology has extended our capability to manipulate subjects of molecular scale. With these unprecedented capabilities, we can directly interrogate and manipulate cells for diagnostic or therapeutic purposes to advance our health care. However, the development of micro/nano devices and the integration of these devices into an engineering system to interface/control a biological complex system are non-trivial. From meters-tall humans to nanometer molecules, physiologically important processes span a disparity of nine orders of magnitude in length scales, which presents significant technical challenges. Therefore, seamlessly integrating nano-, micro- to macro-scale machineries is essential to solve current problems in the bio-medical field [1].

The successful integration of engineering and bio-complex systems requires knowledge in the fundamental difference between the two. Cells, organs and bodies constitute complex systems [2,3,4]; functionalities of a cellular system are manifestations of millions of bio-molecular interactions, and cellular networks change dynamically as they are subjected to external stimuli. In each living cell, the interactions between bio molecules, e.g., proteins and nucleic acids, intrinsically serve as the foundation of extensive networks of signaling and regulatory pathways. However, cellular functionalities emerge from the self-organization of these pathways do not necessarily relate directly to individual bio-molecular interactions [5]. For example, diseases with very different molecular origin may share a common intermediate layer of pathways such as inflammation and immune responses. [6,7]. The resultant pathophenotype may be the same, but the intermediate layer masks the real cause of the diseases. As such, the sheer magnitude of pathway processes and pathway crosstalks presents significant challenges to the straightforward interpretation of them to cellular phenotypic and genotypic outcomes. The functional mapping between the molecular pathway and resultant responses of the bio-system are often indirect as a result of this innate complexity. On the other hand, an engineering micro/nano system is developed based on known design principles and rigid constraints. As such, once the engineering system is developed, it can only perform a specific task and has difficulty in flexibly accommodating agile biological systems.

In order to meet the challenges faced when merging biological and engineering systems, we need to make the next generation microfluidic systems self-adaptive. Micro/nano scale sensors, actuators and decision algorithms will form a re-configurable assembly, in which sensors will measure the dynamic output responses of cells under stimuli. Based on the sensors’ outputs, the decision algorithms will reconfigure the stimuli provided by chemical and mechanical actuators to guide the bio-complex systems towards a directed fate. Hence, both microfluidics and biological systems are “fused” into one “system-in-system” in which the two can adapt to each other and eventually reach a desired outcome. This approach will be particularly effective towards reconciling key challenges that underlie major biological quandaries.

### 1.2. Novel Engineering Systems for Diagnostics and Therapeutics

Since the dawn of MEMS, the same fabrication techniques have been applied to the production of fluidic devices [8,9,10]; to date, more than 15,000 microfluidics-related papers have been published. Driven by the demand for reducing cost of reagents and scaling up measurement of biological assays, microfluidics is becoming one of the backbone technologies for bio-medical industries. Microfluidic systems are particularly suitable for bio-transducers because of their feature size, which can be on the order of microns, the length scale of cells. The matching of length scale offers unprecedented opportunities to explore the unique physical phenomena occurring in the micro world. Microfluidic channels, reactors, molecular sensors and actuators can be automated to move particles/fluid and greatly enhance the efficiency in the detection of disease markers and in the discovery of drugs. The improvements brought by microfluidics leaded to a paradigm shift in bio-medical technologies from centralized biomedical laboratories to a lab-on-a-chip format [11].

Currently, the lab-on-a-chip based disease diagnostics can detect very small number of bio markers; even the detection of a single molecule was demonstrated. Also, high throughput systems with large-scale parallel processing capabilities have become available. These technologies advance the knowledge of the cellular network of signaling and regulatory pathways. As this technology progresses and the cost-per-assay reduced, e.g., $1,000 for the measurement of a human genome, genetic disease diagnosis in clinics will soon be realized [12]. Hence, rational drug design [13,14] and network medicine [15,16,17,18,19,20] were made possible. Despite the benefit offered by high-throughput approaches, these type of bottom-up technologies are inherently expensive and labor intensive, and at times the associated cost for a single diagnosis can be prohibitive for clinical settings. In addition, the exploring phases of disease diagnosis and drug discovery generate large amounts of data. Extraction and interpretation of relevant information can be a major challenge in itself.

To avoid dealing with the explosion of information generated by bottom-up approaches while developing new therapeutics, an unorthodox top-down system level approach, Feedback System Control (FSC), has recently been proposed to reduce the number of experiments by using goal-oriented search [21]. FSC was shown to efficiently hone-in on an optimized drug combination with 10^2^–10^6^ times less number of experiments than a typical high throughput approach. As opposed to collecting all measurable data and trying to find a needle in a haystack, the FSC scheme is a goal-oriented method, which uses the phenotypic outcome to tune the intervention of engineering systems, achieving the system-in-system integration. The fast optimization of a drug cocktail from a large pool of possible combinations has been demonstrated, and it was proven effective in eradicating cancers [22], inhibiting viral infections [23,24] and maintaining human embryonic stem cells [25]. Detailed description of FSC will be discussed in Section 3.2.1.

We will cover trends in microfluidics to probe complex bionetworks and provide a discussion of the importance of interfacial technologies to link microfluidic systems with the information rich bio-complex systems for advancing the diagnostic and therapeutic capabilities. However, this paper does not intend to give an extensive review of existing bio micro/nano transducers. Readers are encouraged to find information on microfluidics [26,27,28,29,30,31,32,33,34,35], transducers [36,37,38,39,40,41,42,43,44,45] and drug discoveries [46,47,48,49,50] in the other excellent review papers.

## 2. Progresses in Diagnostic Systems

### 2.1. Lab-on-a-Chip (LOC) Based Point-of-Care Diagnosis

A lab-on-a-chip diagnostic platform consists of sample preparation fluidic circuitry and sensors. The bio molecules of interest need to be collected from bodily fluids or tissue samples, e.g., tissue biopsy, blood, urine, and saliva, and delivered to the sensing sites for determining concentration levels of markers. The fluidic processes involve moving and stopping fluids/particles, mixing, and separation. Force fields, such as hydrodynamic, electrokinetic, surface tension and optical tweezers are used for accomplishing the fluidic processes [51]. The manufacturing techniques of such fluidic devices and sensors are fairly well established [32,34,52]. The challenges remain in the integration of devices into an efficient system and in the modifications of surface molecular properties. Properly modified surfaces can avoid fouling in the fluidic circuit and reduce the noise level of sensors.

Abnormal expressions of nucleic acids and/or proteins can be used as markers to determine diseases. MEMS based sensors for these biomarkers have been an active research field for the past decades [53]. The limit of detection (LOD) and specificity are the performance measures of sensors. Signal to noise ratio determines the LOD; consequently, reducing noise is a key challenge. For example, with proper sensor surface treatment, a LOD of 10 aM is established for IL-8 mRNA in saliva. The typical sample volume used in the detection is 4 µL, thus the LOD reaches only 25 target molecules without PCR amplification (40 yoctomole). A LOD of protein of 100–200 fg·mL^−1^ is also achieved in the literature [54]. In terms of specificity, molecular beacon probes can detect nucleic acids with a single base mutation [55]. Sensors capable of detecting molecules in live cells will increase the knowledge of live cell network activities and have promising potential for future development [56].

### 2.2. High Throughput Sensor Systems

**Figure 1:**
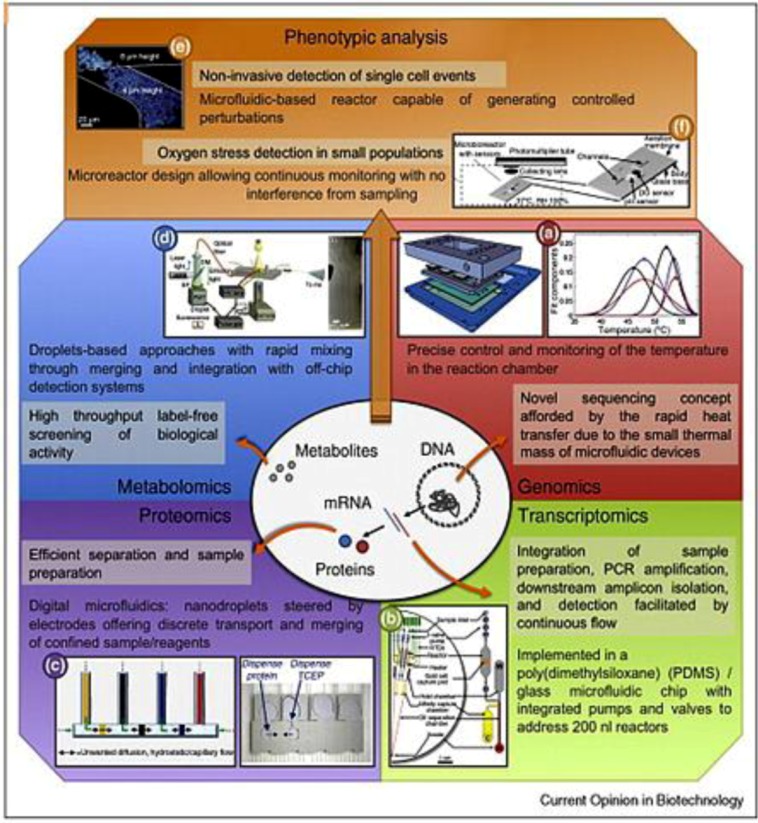
Different high throughput analyses carried out in microfluidic devices, highlighting the advantages of microfluidic approaches to gather information for omics. Adapted with permission from [61], Copyright (2010) Elsevier Ltd.

Biological systems are governed by the flow of information through DNA, mRNA, and proteins. Proteins function as the major machineries in maintaining the vital functions of cellular organisms, and they also regulate the transcription events in DNA. Furthermore, there are also other processes involved in maintaining cellular functions, including epigenetic regulations and post-translational modifications. These processes can be visualized by a graph of an integrated biological network, involving nodes representing entities, such as proteins and mRNAs, and edges representing their physical interactions. Disturbances found in any part of the flow of information inside the network are often the cause of diseases [19]. These disturbances may be caused by genetic mutations, environmental effects or infectious agents. The disturbances will then propagate and affect other parts of the cellular network [18]. To capture the affected states in space and time, we need to monitor many, if not all, nodes in the network simultaneously. Therefore, it is an inevitable trend to shift the understanding of disease from a single defect in the pathway to the totality of the systems network, in other words, the omics. The implication of the omics goes well beyond the science of disease origin and the search of biomarkers. In fact, a single biomarker is a concept associated with reductionism; this kind of diagnostic practice often fails to define disease unequivocally and has little predictive power in identifying pre-disease state [16]. It was during the last two decades that an explosion of information in the areas of genomics, proteomics, and metabolomics took place; the shift in disease understanding toward network thinking was enabled by the successful implementation of high-throughput systems, especially the microfluidics systems as illustrated in Figure 1.

Microfluidic systems are particularly suited for uncovering disease dynamics and multiparameter disease diagnostics for several reasons. Frist, the microfluidic technologies provide transducers that match cellular scale. In most transduction processes, matching of length scales is a fundamental requirement; it seems natural to adapt the traditional biochemical techniques to a smaller length scale. Second, the high-throughput capability of microfluidics is capable of providing the amount of data required by systems biology and network medicine, soon reaching petabyte range [16,57]. The manufacturing technologies of microfluidic systems are derived from the semiconductor batch processing method in which millions of transistors are put together to perform highly parallel tasks. Multilayer microfluidic devices use flexible mechanical valves and pumps to drive and direct the working fluid in channels, and they can easily be fabricated in a densely packed configuration of arrays [58]. Third, microfluidics can significantly reduce the consumption of reagents. The high throughput biomedical assays are mostly performed by using traditional microtiter plates aided by liquid handling robot. These formats consume microliters of expensive reagents, while each well in a typical microfluidic device stores a volume lower than a nanoliter. The saving of reagents by more than a factor of 10^3^ will enable tasks that are currently prohibitively expensive to handle through traditional methods [59]. Fourth, the large surface-to-volume ratio in microfluidics can facilitate multiple processes in biomedical assays [58]. For example, by taking advantage of the high surface-to-volume ratio offered by microchannels, Chen *et al.* were able to permeate more oxygen into the channels as compared to bulk culture and facilitated bacterial growth; the antimicrobial resistance was probed in 2 h instead of several days [60]. With the tremendous processing power, microfluidics is becoming an indispensable tool for future high-throughput diagnostics in genomics, proteomics and metabolomics.

#### 2.2.1. Genomics

The human genome project spurred a public frenzy and revealed to the public the potential implication of deciphering the code of life [62], and it impacts diagnostics in three aspects. First, having genome sequences can help define the complete list of all human genes and proteins, which can yield new drug targets for novel treatments. Second, the process uncovers common variants of the genome, which aids determination of hereditary factors in diseases. Third, genetic maps can guide experiments of structural genomics, thus enabling large-scale experiments of protein crystallization to determine protein structures [63]. More recently, next generation sequencing and single molecule sequencing technologies have promised to bring low cost genomic data and make genome-profiled medicine a reality. Whole genome sequencing can be applied in molecular diagnostics to analyze genomic disorders such as Mendelian disorders or common diseases in which the risk for disease development is modulated by multiple genes [64]. Genomic study techniques, such as genome-wide association studies [65], high-density genotyping, and exome sequencing [64], are only made possible by cost effective genome sequencing technologies [59,66,67,68].

One of the main focuses in genomic research is genotyping; in particular, single nucleotide polymorphism (SNP) which accounts for 80% of the variation between two individuals [69]. SNPs can cause structural changes in proteins, alter drug metabolism, and change gene regulations. The driving force behind the SNP studies is the identification of SNPs as disease markers, which is implemented by comparing the SNPs between the diseased phenotype and controls. It is estimated that around 1 million to 0.5 million SNPs would be required for a whole genome mapping of each individual patient, and 1.5 million SNPs are required per day for a reasonable pathological study (the variation arises from genetic and environmental heterogeneity of the population) [70]. In order to collect such a huge set of data, a platform needs to be robust, high throughput, and low cost. Microfluidic technologies developed for the large-scale analysis of SNPs suit this purpose very well. Recent developments in the detection of SNPs improved the existing microarray technology and use probes immobilized on microbeads to allow faster diffusion of the probes and raise the surface-to-volume ratio to increase the sensitivity and speed up SNP assays [71,72]. The advent of second generation sequencing marks down major cost of whole genome sequencing, and it expands the number of applications for the microarray technology. Sequencing coupled with microarray genotyping and bioinformatics studies have already allowed large-scale study of the SNPs [73,74]. Further cost cuts in single molecule sequencing technology will eventually pave the way for personalized diagnostics and therapeutics. 

On top of genetics, epigenetic factors are equally important to consider. Epigenetic signals regulate gene expressions and determine cellular phenotypes; some epigenetic signals are shown to be important in the prognosis of diseases [75,76]. In the post-genomic era, epigenetics will become the fastest growing field of study in molecular biology. For example, DNA methylations of the tumor suppressor genes were shown to be important early diagnostic markers to predict carcinogenesis. Recently, Wang *et al.* reported performing early diagnosis for ovarian cancer as a proof-of-concept that microfluidics can facilitate epigenetic studies [77]. Aline *et al.* use micro-wells patterned by soft-lithography and meniscus force to trap and elongate single stranded DNAs, allowing high throughput epigenetic mapping [78].

Transcriptomics concerns the catalogue of all human RNA transcripts and the gene expression profiles under specific biological conditions, and it is related to the diagnostics of many diseases, including lung cancer [79], leukemia [80], breast cancer [81], acute ischemic stroke [82], to name a few. The studies of gene expression levels were traditionally accomplished with microarrays, but the toolbox for transcriptomics is rapidly expanding especially with the recent introduction of the next generation sequencing technologies. RNA-seq uses the deep sequencing technology and can directly quantify the expression levels of cDNAs of the transcripts as well as obtaining the sequences of the transcripts [83,84]. Due to the additional information of sequences, RNA-seq does not rely on preexisting genomic data and can study the subject without the complete genome. Also, with the sequences of the transcripts at hand, RNA-seq can also provide information for the connectivity between exons. As for the accuracy for the methods, compared to that of microarrays, RNA-seq has no upper bound for the dynamic range of transcripts and is less prone to background noise. Despite the clear advantage of RNA-seq over the traditional microarray technology, microarrays will more likely dominate the clinical setting in the short term for their relative maturity and lower cost. 

**Gene Sequencing:** The rapid advancement of gene sequencing technology is the key enabler in changing the way diseases are classified and diagnosed [33,74,85,86,87,88]. The original Sanger sequencing uses chain termination chemistry to introduce random stops in the transcription events. Then, the collection of different length DNA oligos (which are labeled with the chain terminating dideoxyNTPs) are subsequently analyzed by capillary electrophoresis (CE). There are substantial efforts being made in the research community to miniaturize electrophoretic sequencing, enabling faster processing time and reduced reagent consumptions. Multiple processes for the sample preparations can be improved, including real-time PCR amplification, template purification, reaction of the extension of DNAs, and enrichment of DNA oligos. Despite the effort to adapt electrophoresis sequencing to microfluidic technology, the throughput still does not match that of the cyclic array sequencing. Even though Sanger sequencing has lower throughput compared to next generation sequencing it still remains as a good option for de novo sequencing of complex new genomes and low scale applications because of its long read lengths and flexibility in scale [89]. In the human genome project, CE is performed in a 96 or 384 channel format. With 3 billion dollars in public funding and an international consortium of labs, it still took 15 years to complete the project [90,91].

Second generation sequencing uses shotgun sequencing with the cyclic array method [66,92]. The cDNA is first randomly fragmented to create a library of single stranded DNA oligos. These fragments are then ligated with some common adaptors and immobilized to a planner substrate to form a 2 dimensional pattern of arrays. These single stranded DNAs are then amplified on-chip to form PCR colonies such that each colony on the substrate only represents one oligo in the library. Fluorescent labeled nucleotides are then extended one by one onto the single stranded colony by solid state chemistry, and images taken at each step record the bases incorporated into each colony in one cycle. The process iterates until all the oligos are sequenced. Finally, the acquired shotgun sequence is reassembled in-silico to form the complete sequences of the sample. The true power of the 2nd generation technologies lay in the great density of colonies one can form on a planer substrate. The major drawback for the second generation sequencing is the short read length of oligos and time-consuming washing steps.

**Figure 2:**
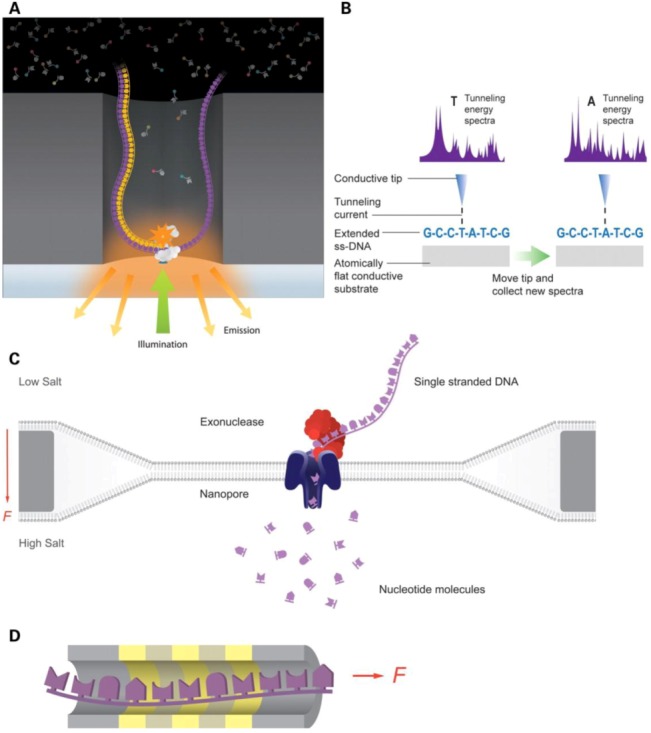
Third generation sequencing technologies. (**a**) Pacific biosciences technology, a DNA polymerase performs the synthesis while the base additions are detected with labeled phosphonycleotides. (**b**) Reveo technology, Single-Stranded DNA (ssDNA) molecules are stretched and further analyzed by Scanning Tunneling Microscope (STM). (**c**) Oxford nanopore technology, nucleotide translocation across a nanopore is measured. (**d**) IBM’s DNA transistor technology, individual bases of ssDNA are detected as they pass through a transistor channel. Adapted with permission from [59]. Copyright (2012) Oxford University Press.

Currently, the major focus for the third generation sequencing (TGS) is to shrink the sensors down to molecular scale [59,68,93,94], and thus is radically different from the second generation sequencing platform. The TGS techniques read signals generated from direct physical interactions of single molecule DNAs with sensors, as shown in Figure 2. Two kinds of TGS are the most promising in the field: one was enabled by nanopores [95,96,97,98] and the other by single molecule sequencing by synthesis [93,99]. Sequencing with nanopores takes advantage of the electric signals generated when DNAs are passed through hollowed nanostructures, which can be silicon based or protein based. The shape and size of the hollowed nanostructure is important for the signals and noises generated from electrical interactions between the structures and DNAs. For example, one of the most successful TGS is the Oxford Nanopore technology. In this technology, a hollowed nanostructure is fabricated from a genetically engineered α-hemolysin with the pore size modified by attachment of a synthetic cyclodextrin. Such nanopores are embedded in a lipid bilayer membrane, and an external electric field is applied across the membrane to induce DNAs to translocate through the nanopores. Electrical signals are generated in real time during translocations, allowing the sequences to be rapidly determined. The successful implementations of the TGS technologies relied on advanced knowledge of nanoscale phenomena. Due to the distinct physical principles involved, the applications of TGS technologies can go beyond sequencing and extend to the identification of epigenetic modifications [100], high throughput transcriptomics [101], and genome-wide translation studies [102].

**Microarray:** Microarrays have been a major platform for the parallel analysis of a mixture of DNA or RNA nucleic acid samples, with a variety of new microarray and chip devices and up-stream sample processing systems being developed and commercialized every year. The common applications of these devices are the analyses of gene expression level, or detection of single nucleotide polymorphisms (SNPs). In addition to the genomic and molecular biologically related applications, microarray systems are extensively used for pharmacogenomic research, disease diagnostics, and forensic identification purposes [103]. In General, a DNA microarray consists of single stranded DNA probe molecules immobilized on a solid substrate and patterned as an array of microspots. Sample solutions, containing marker modified ssDNA/RNA, was transported to microarray by either a bulk or microfluidic liquid handling method; the ssDNA/RNA was allowed to hybridized to the probe and the unmatched strands are washed away. The time limiting factor in the hybridization process is usually diffusion, which dictates the speed at which the analyte migrates toward the immobilized probes. The diffusion time scale is long compared to the kinetic time scales associated with molecular binding events. The low diffusivity of DNA (D ~10^8^ to 10^9^ (cm^2^·s^−1^)) arises from its molecular length and conformation, making a typical hybridization step hours long. The excessive time required for hybridization presents a roadblock for high-throughput screening and sample-in-answer-out applications, and is an active research problem which the microfluidic technology can potentially solve [30].

Recent advancements in microfluidic hybridization techniques can significantly reduce the incubation time. There are two means to shorten the hybridization event: one is enhanced convection through active agitation and the other is to increase the surface-to-volume ratio. In the convective method, sample solutions are confined in microfabricated channels and flow through the area of probes. The confined nanoliter structures and high surface-to-volume ratio in microchannels greatly enhanced the sensitivity compare to that of the bulk solution method. To generate convective flows, different pumping techniques have been developed, and these include electrokinetic flow, vacuum or syringe pumping, and centrifugal force. Except for the electrokinetic flow, which can introduce complications in manipulating ionic mixtures, the others are contact flows which are more flexible because they do not consider the physicochemical properties of the solute [104]. In the second method, the surface-to-volume ratio is increased by using microchannels or microbeads. The microbead technique shows great promise [10,105,106]. Various microbeads can be used as low-cost solid supports, including latex, silica, and gold colloidal nanoparticles or quantum dots; they are in some cases functionalized with oligomers and fluorophores to be tracked and addressed [107]. By comparing a pixel area of 1 μm^2^ for traditional DNA microarrays to a 100 μL sample of 1% microbeads, the beads offer a capturing area larger by eight-orders of magnitude [108]. With the incorporation of microfluidic technology, microarrays continue to improve in sensitivity and speed and are becoming a more economical research tool.

#### 2.2.2. Proteomics

Proteomics studies the *totality of proteins* that are present in a given organism. Because proteins are the key governors of cellular function, monitoring and comparing the state of the proteome in normal and diseased cells will be important in unlocking the underlying mechanisms of disease phenotypes and in finding the best biomarkers for diagnosis and targets for therapy. However, proteomics studies are even more difficult than genomics due to the lack of a PCR analogue to amplify the already scarce source of proteins. The human proteome is the totality of proteins that are present in the human body, including proteins in different types of cells. All these proteins are encoded in the human genomic DNA but undergo post-translational modifications (PTMs). There are about 30,000 genes in the human DNA, but there are estimated to be around 300,000 types of proteins. In addition to the complexity of the proteome compositions, the expressed levels of proteins vary over several orders of magnitude, which present a significant challenge when the proteins of interest are scarce compared to the background. For example, albumin and immunoglobulins account for 55% of all proteins present in human plasma, but the protein markers of interest are a million to a billion times lower in concentration [109].

Despite the challenges faced by proteomics study, there are still strong reasons why we need to study proteins directly. Firstly, mRNA microarrays are found to correlate poorly with the protein expression levels [110]. Second, protein-protein interactions like PTMs can only be studied by direct protein experiments. Although the information contained in genomics is supposed to encode the activity of proteins, a method does not currently exist to decode this information from genomics directly. 

The analytical methods of proteomics have not moved as quickly as genomics research due to the following reasons: first, unlike DNA, which does not degrade in response to a large range of environmental perturbations, proteins are less tolerant to change in environmental factors such as temperature, PH level, and salt content. The denaturing of proteins restricts the use of solvents in assays and poses technical challenges in every stage of protein analysis. Second, there is no PCR analogue that exists in protein analysis, meaning that the sensing method must be sensitive to low concentrations of protein.

**Figure 3:**
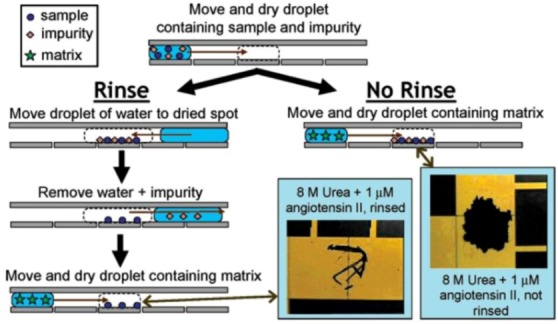
Digital microfluidic (DMF) systems are proven to be capable of handling the sample processing in Mass Spectrometry (MS), Electrowetting on Dielectrics (EWOD) combined with Matrix assisted laser desorption/ionization (MALDI). Reprinted with permission from [112], Copyright (2005) American Chemical Society.

**Microfluidics and Mass Spectrometry Integration:** One of the major uses of microfluidics in proteomics is to tether with mass spectrometry (MS) to do sample processing for the analysis. The limited sample starting amounts, the multistep analyses, and the operating cost of the expensive MS equipment necessitate a reliable microfluidics system to perform the chosen separation strategy. MS measures the mass-to-charge ratio by separating analytes with a Lorentz force. Due to the high sensitivity and robustness of MS, it is still a method of choice even though the instrumentation remains macro scale. The way microfluidics is integrated with MS is strongly dependent on the ionization employed. A popular on-line method, electrospray ionization (ESI), is tethered directly to the microfluidics exit while the analyte is ionized in real time. Yun *et al*. demonstrated the use of plastic microfluidics integrated with ESI that improved the detection sensitivity and reduced dead volume in the original format [111]. Matrix assisted laser desorption/ionization (MALDI), a major off-line method, uses microfluidics to pre-deposit sample matrices with analytes spots, and can be off-line processed by laser ionization. As highlighted in Figure 3, Digital microfluidic (DMF) systems, is capable of automating the sample processing in MALDI, and reducing contaminations of analytes [112,113,114]. For a detailed review, please refer to [30,115,116]. 

**Figure 4:**
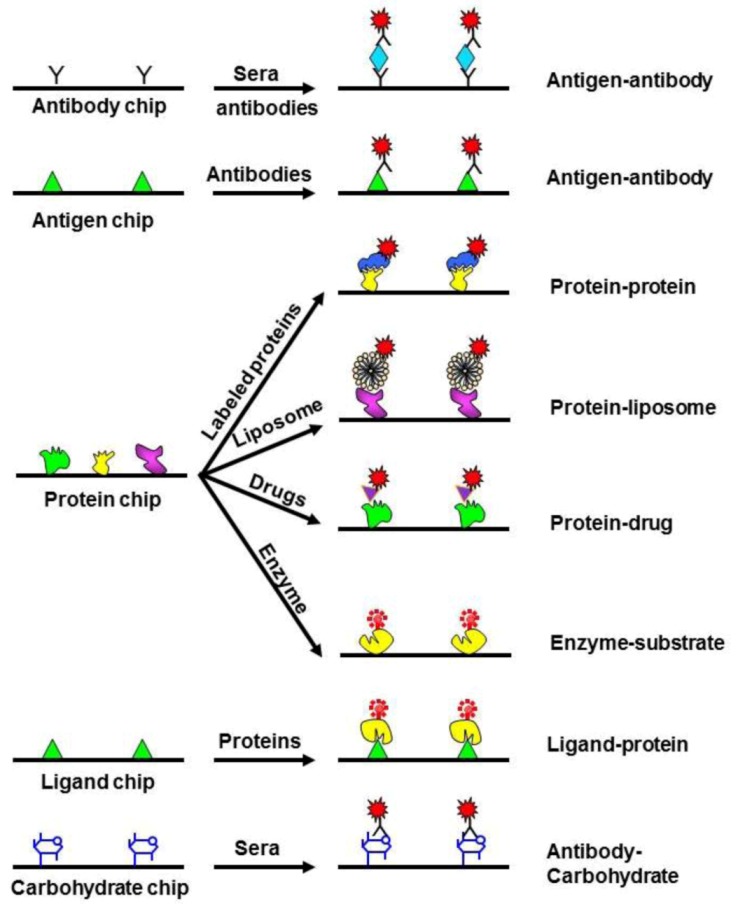
Applications of protein arrays. Analytical microarrays consist of a high-density array of affinity reagents, such as antibodies or antigens; they can also involve the use of ligands or carbohydrates. On the other hand, functional microarrays are made by immobilizing high numbers of purified proteins. Reprinted with permission from [117], Copyright (2003) Elsevier Science Ltd.

**Protein Microarray:** Protein arrays can impact our understanding of protein structure and function in the same way that DNA arrays provided insight into gene expression and regulation. There are commonly two types of array modality: functional and analytical protein microarrays, as illustrated in Figure 4 [118]. Functional protein microarrays are usually applied to analyze a genomic set of proteins. In this type of array, individual proteins are separately spotted on a surface such as a glass slide and then analyzed for activity. This approach has the potential for rapid high-throughput analysis of large collections of proteins and cellular proteomes, and promises to transform the pharmaceutical industry. A second type of protein microarray is the analytical microarray. Here, a genomic set of protein-specific ligands such as nucleic acid aptamers, antibodies, or chemical probes are spotted on a microarray, and then the proteins in an extract can be quantified in parallel by binding extracted proteins to the microarray. The scientific community is starting to realize the potential of analytical protein microarrays to monitor protein expression on a proteome-wide scale and in medical diagnostics. For example, protein microarray was recently applied in the diagnosis of complex disease such as acute myocardial infraction [80], autoimmune disease [81], and cancer [82]. A critical step in generating a practical protein array has been the development of general methods for arraying a large set of proteins without denaturing them and at enough density for detection of interactions.

**Figure 5:**
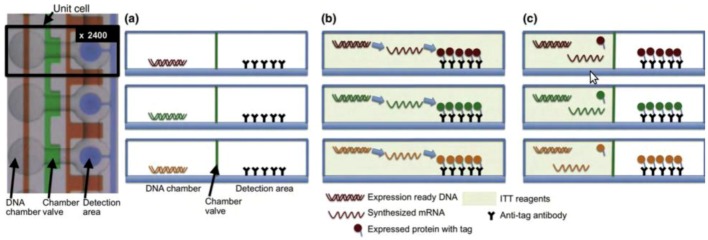
DNA to protein arrays microfluidic platform. (**a**) DNA templates are enclosed on each unit chamber, while the detection area contains immobilized antibodies. (**b**) To generate the DNA to protein array (DTPA) the device is loaded with the *in vitro* transcription/translation (ITT) reaction mixture, allowing for protein synthesis to occur. (**c**) The detection area is washed to remove any ITT leftovers, and the protein array is now ready for further analysis. Reprinted with permission from [123], Copyright (2010) Elsevier Ltd.

Recent technological developments have addressed the difficulties associated with purifying a large set of proteins for array construction. The invention of DNA to protein arrays (DTPAs) using *in vitro* transcription/translation (ITT) circumvents many of the difficulties associated with cloning, expressing, purifying, and spotting of proteins, and is depicted in Figure 5. Currently, the *in situ* or on-chip protein array methods use cell free expression systems to produce proteins directly onto an immobilizing surface from co-distributed or pre-arrayed DNA or RNA, thus enabling protein arrays to be created on demand [119]. Integration of DTPAs and microfluidics has been demonstrated for the synthesis and characterization of S. pneumoniae proteins [120] and synthetic transcription factor mutants [121]. Besides advancement in the chip patterning, several detection methods were also developed to detect interaction of proteins with proteins and nucleic acids; these methods include FRET, FCS, MITOMI, SPR, and Nanowire. The integration of novel and existing methods for measuring molecular interactions allows proteins to be characterized quantitatively and with better sensitivity. The integration of DTPAs and novel detection mechanisms into a single microfluidic device platform is a significant step toward automating these methods, and increasing throughput in protein biochemistry. For more in depth reviews of protein microarrays, the readers are encouraged to refer to the following [118,122,123].

#### 2.2.3. Metabolomics

**Figure 6:**
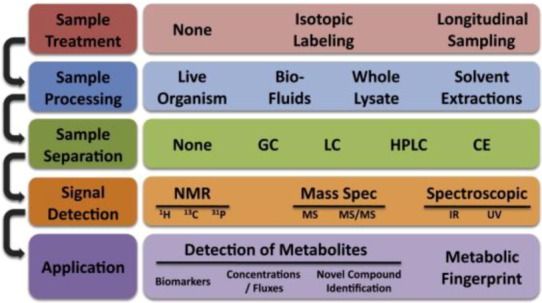
The typical workflow for single cell metabolomics analysis. Multiple experimental steps are involved in the metabolomics study, each one with different possible methodologies. Reprinted with permission from [128], Copyright (2010) Elsevier Inc.

Metabolomics is the study of the totality of metabolite expressions as a snap shot or as a dynamic course in time. At the single cell level, metabolites play a critical role in intercellular communications, energy balance, osmoregulation, membrane dynamics and other cellular activities. Metabolomics can provide useful insight into the state of diseases [124]. Because metabolites can be extracted easily from cells, blood or serum, they can help to develop noninvasive diagnostics technologies. Biomarkers derived from metabolomics studies have been proven sensitive and efficient in helping stratify patient population and evaluate prognosis. For example, Sreekumar *et al*. identified a set of metabolites that can be used as biomarkers to distinguish the function of a normal prostate from that of a prostate cancer patient [125]. They found that sarcosine levels correlate very well with cancer progression and can be used as biomarkers for noninvasive diagnosis [125]. Metabolites are usually analyzed noninvasively by nuclear magnetic resonance spectroscopy and *in vitro* by traditional chemical analytical technologies, such as liquid chromatography (LC) or gas chromatography (GC), coupled with mass spectrometry; the typical workflow of metabolomics is covered in Figure 6. For a detailed review of metabolomic processes, please refer to [126]. Microfluidics were introduced only recently into the study of metabolomics. However, just as in proteomics, microfluidic systems have not displayed their full potential in metabolomic studies and have mainly served only as platforms for sample preparations [127].

#### 2.2.4. Single Cell Diagnostics

Traditional bioanalytical techniques usually take up a group of cells to enrich the analyte content to a detectable range and record the ensemble average of measurements. Recently, with the advancement in the sensitivity and ability to multiplex in assays, single cell analysis is beginning to revolutionize traditional batch format experiments by recovering the hidden information arising from the heterogeneity of populations of cells. Cell samples taken from tissue samples contain many different phenotypes of cells. Furthermore, cells taken from culture of same type of cells also contain different expression profiles, representing multiple levels of heterogeneity [129]. The heterogeneity of cells is important because the value of interest often does not follow a simple statistical distribution, so an ensemble average is not a representation of any individual in a population, as exemplified by the mRNA expression of GAPDH of Jurkat cells after siRNA knockdowns [130]. Also, the environmental changes are likely to induce redistribution of gene regulatory network states in the cell population, in which the dynamics can only be traced with single cell analysis [18]. Recently, with the advancement of sequencing capability, intra tumor heterogeneity, which dictates tumor evolution and adaptation and disrupts therapeutic strategy planned with single tumor biopsy sample, has just begun to surface [131].

## 3. Directing a Bio Complex System toward desired Fate for Therapeutic Purpose

Bio-molecule based disease diagnostics is a challenging endeavor. The major goal of diagnostics is to find an array of bio markers which can accurately determine a type of disease. Similarly, the goal of drug therapeutics is to discover chemical(s) which can tune network dynamics of the human body and guide cells, tissues, organs, and the whole organism toward a desired fate, such as restoration to a healthy state, or eradication of infectious agents. Two fundamentally different philosophies, the bottom-up and top-down approaches, currently govern how the strategies of therapeutics are determined.

### 3.1. Bottom-Up Approach of Drug Discovery

Most of the drugs in use today were FDA approved before the 1960’s and were developed by trial and error [48]. After 1960s, mechanism based drug search became the main stream of new drug developments. Bottom-up reductionist approach starts from the molecular level, nucleic acids or proteins, to establish their chain reactions, pathways, and find a drug molecule to affect the pathophenotype. The difficulty of this type of drug discovery is to design a molecule which can bind to a target and has therapeutic effects but not bind to other cellular molecules to cause toxicities [132]. The huge number of possibilities to sort out a proper drug molecule and to avoid unwanted interactions with cellular molecules other than those targeted make the task formidable.

#### 3.1.1. Targeted Therapy and Rational Design of Drugs

Rational design of drugs follows the bottom-up approaches and identifies disease mechanisms by searching through aberrant molecular machineries. With experimental high throughput screening and numerical modeling, a molecule complimentary in structure and electrical charge to the disease causing cellular molecule is designed and is used to treat the disease. Efficient docking of drugs to targets or ligands requires accurate knowledge of the 3-D molecular structures and charge distributions. Large-scale computer simulations and fast progresses in parallel processing fluidic instrumentation have facilitated the tasks of searching for the desired drug molecules from a large pool of possibilities.

Targeted therapy and rational drug design oversimplified the interconnected nature of diseases, and is in accord with the reductionist paradigm which drove medical practice in the modern era [16]. The fact that most disease phenotypes cannot be reversed by a single node or single edge treatment in the network best exemplifies the difficulty faced by the reductionist paradigm. Under the reductionist framework, biological information was trimmed into a single mechanistic pathway, and the associated treatment was reduced to usually a single component in the pathway. With ingenious ideas and diligent work, targeted drugs have made significant impacts in treating cancers and many other diseases during the past several decades. A rationally designed drug is successful in coupling with monogenic diseases, but is not sufficient for certain diseases that are more complex in nature. In addition, robust biological systems can often develop resistance by bypassing the targeted pathway, which reduces the efficacy of treatments. 

#### 3.1.2. Network Medicine—A Bottom-Up Approach

Network medicine is an emerging field which incorporates concepts in systems biology and takes into account the structure and dynamics of a complex network in the interpretation of disease. Cancer, aging, Alzheimer’s disease are all complex diseases which cannot be reduced to a single molecular cause, and can be understood more easily under the context of biological networks. Instead of pointing the molecular cause of a disease to a single pathway, a disease is interpreted by the concepts of network medicine as network perturbations in a dynamic system, so diseased states can be defined more precisely [16,18]. Useful clinical and biological applications can be derived from network approaches. The increased understanding of the interconnectedness of cellular network will lead to understanding of the causes of disease pathogenesis, identification of more precise sets of biomarkers for diagnosis, and eventually therapeutics.

Recently, combining the concept in network biology and polypharmacology, multi-target therapy is gaining traction as the main strategy in drug discovery [133], and has been demonstrated successfully in systems such as HIV infection [134], and cancer therapy. Combination of multiple drugs has many advantages over single drug therapy: (1) drug combinations enhance the potency of the therapy by taking advantage of the drug synergistic or additive effects; (2) the synergies among many drugs may reduce the dosage levels, which usually imply low toxicities and side effects; (3) it can avoid emergence of drug resistance by combining drugs with minimal cross-resistance [135]. However, compared to disease identification, the application of network medicine concept directly to multi-target therapeutics still suffers the inherent difficulty of the bottom-up approaches. 

The network medicine approach [17] of choosing drug targets is based on the knowledge derived from genomics, proteomics, pathways and pathways’ interactions through the network connections. This type of bottom-up approaches can provide fundamental understanding of the disease-causing and drug-interaction mechanisms, only if the complete molecular activities and pathway information are available. Given that the datasets from genomics to proteomic and from pathways to phenotype are currently far from complete, the application of bottom-up approaches in the clinics would still be too difficult and expensive. We surmise that the wide spread applications of bottom-up approaches can only be achieved once reliable databases, robust high-throughput platforms, and petabyte processing capabilities are met.

### 3.2. Top-Down Goal Oriented Approach of Combinatorial Drug Optimization

Treating disease with multiple synergetic drugs has the advantage of high efficacy and low toxicity. The bottom-up network medicine based drug selection method needs detailed molecular and pathway information, which is hard to get. The network medicine based drug selection method can support the choices based on knowledge of the network topology. However, this approach cannot provide the proper dosage for each drug used in the combination. Dosage ratios of the drugs are very important in multiple drug therapy; the efficacy can be significantly affected by the dosage ratios.

An unorthodox top-down system approach to optimize the combinatorial drug-dosage has been demonstrated. In the FSC approach, we do not rely on the cellular molecular and pathway information; we instead take a black-box approach. Based on the cellular system outputs and phenotype, we search for the best performing drug-dosage combinations.

#### 3.2.1. Feedback System Control (FSC) Based Combinatorial Drug Optimization

The ultimate goal of therapy is to cure the disease. In order to control and eventually reverse the pathophenotypes, the Feedback System Control (FSC) technique was developed to search for the optimal dosage for combinatorial drug therapy. The phenotype was taken as parameters to construct an objective function, in which the goal is measured. The objective function sits in an engineering feedback loop, which is used to direct engineering systems to tune the external stimuli. In the top-down approach, the bio system is driven to the desired state without measurement of the detailed molecular activities inside the cell, thus, avoiding the overloading of information common in bottom-up approach.

**Figure 7:**
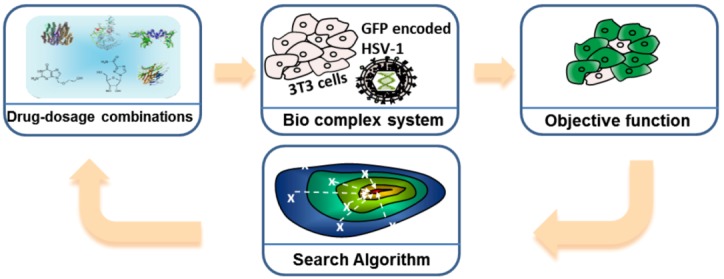
Feedback system control (FSC) scheme applied to the optimization of an Herpes Virus Simplex 1 (HSV-1) viral infection drug treatment. Drug combinations serve as the input stimuli for the biological complex system, the infected 3T3 cells; the virus attempts to infect normal cells, and the drug combinations try to inhibit virus infection. The objective function, or output, measures the efficiency of the drug combination. The search algorithm reads the output and suggests new drug combinations to be tested, and these combinations improve with each iteration, leading to fewer infected cells. Adapted with permission from [23,24], Copyright (2012) Ding *et al.*, publisher and licensee Dove Medical Press Ltd.

FSC consists of four modules as shown in Figure 7. Let us take the Herpes Virus Simplex 1 (HSV-1) infection experiment as an example [24]. The first module is the input stimuli, e.g., the drug-dosage combinations. The second module is the bio-complex system of interest, e.g., virus and host cell. The third module is the objective function, e.g., the percentage of infected host cells, and/or toxicity/side effects. The fourth module is the search algorithm, which provides the next set of stimuli and dosages for directing the biological complex system toward the desired state.

If the dynamics of the plant, e.g., the cellular activities, are known in full detail, analytical modeling of the entire feedback loop can be performed. The FSC approach aims to circumvent the need for detailed information of the system of interest, so that *a priori* knowledge of the bio-system is not required, *i.e*., treating the cell as a black box. In the first test, the inputs are arbitrarily chosen drug-dosage combinations. The cells will respond to the input stimulants and exhibit system outputs. The system responses are usually non-satisfactory because the inputs are not optimized. Based on the first drug-dosage combination and the outputs, the search algorithm will come up with new and perhaps better combinatorial stimulants and feedback to the bio system. The feedback loops will be continued until the desired phenotype occurs.

An optimization of a drug-dosage combination that involves M stimulants at N levels generates a pool of N^M^ total testing cases. A screening of the entire pool, N^M^ cases, is prohibitive. In other words, if the feedback loop approach cannot find the optimal drug-dosage in a small number of iterations, then the FSC technique (even bypassing the need of detailed dynamics of the plant) still will not be a feasible method for meeting our requirements. 

In the experiment of inhibiting HSV-1 infection, we used FSC technique to search for the optimal drug combination from a pool of 1,000,000 combinations [24]. Only about 12 iteration loops were needed. The search started with 6 drugs. Only three drugs with much lower dosages compared with single drug therapy were needed to achieve close to 100% inhibition.

FSC is a black box or modeless approach. Only a small number of iterations in the feedback loop are needed to find the most potent drug combination. Counts of 10–15 iterations to hone-in on the optimal drug cocktail have also been achieved in eradication of cancers, maintaining human embryonic stem cells and in inhibiting viral infections [22,25]. The key finding in the FSC-based studies is that the system’s response surface to the drug stimuli is very smooth. Therefore, not many iterations are needed to reach the best performance in the high dimensional (multiple drugs) space. Due to the smoothness of the response surface, the choice of search algorithm is not very sensitive in affecting the number of iterations needed to reach optimal combination in all our tested models. In addition, the low dosage of each drug in the cocktail is a result of the synergetic effects among drugs. 

### 3.3. Microfluidic Based Instrumentations for Analyzing Cellular Systems

#### 3.3.1. Micro Fluorescence-Activated Cell Sorting (FACS) for Sample Preparation

**Figure 8:**
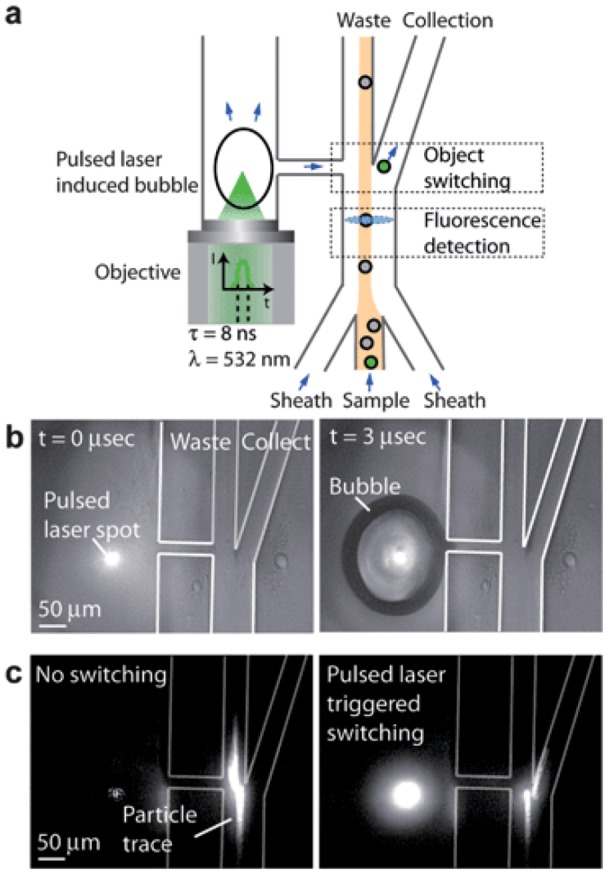
Schematic of the pulsed laser triggered high-speed microfluidic Fluorescence-Activated Cell Sorting (FACS). (**a**) When a fluorescent sample flows through the fluorescence detection region, a laser pulse is triggered and induces a cavitation bubble in the pulsed channel. The bubble expansion produces a high-speed liquid jet, directing the sample towards the collection channel for sorting. (**b**) Cavitation bubble generated by the focused pulsed laser beam in the microfluidic cell sorter at t = 0 µs and t =3 µs. (**c**) Fluorescent particle switching. When the fluorescence activated switching is not in use, the particle is directed towards the waste outlet. When the fluorescence activated switching is being used, the particle is directed towards the collection outlet. Reprinted with permission from [137]. Copyright (2012) Royal Society of Chemistry.

Disease is a system problem and needs to be studied at the system level. Cells are the first level of this system. For instance, if we can identify and collect cancer cells from blood, then we may be able to detect and to treat the cancer at an early stage. Microfluidic sample preparation processes offer an emerging technology for sorting rare cells from bodily fluids. Flow cytometry and fluorescence-activated cell sorting (FACS) are powerful instruments in sorting minute amount of cells with specific membrane markers from large number of red and white blood cells [136]. The commercial FACS can sort at the speed of 10,000 switching events/sec. However, the switching rate of microfluidics based FACS was a few orders of magnitude slower until a high speed pulse laser triggered micro bubble switch was invented (Figure 8) [137]. The pulse laser induces a bubble near the elastic fluidic channel. The rapid deformation of the channel wall can change the direction of the motion of a cell marked by fluorophore conjugated with a specific cancer membrane antibody. The switching rate matches or is even higher than that of large commercial products. In addition, cells are maintained in a friendly buffer environment so that high cell viability can be obtained. Furthermore, only a very small amount of fluid volume is needed for processing in a microfluidic system. This is particularly important in clinical applications. In most cases, the biopsy sample from patients is so small that only microfluidic devices can offer efficient sample preparation. 

#### 3.3.2. Dissecting Network Responses through Phosphorylated Protein Analysis

It is well known that a cellular response to a stimulus involves several actions, particularly protein modification through post-translational processes. These include protein cleavage, protein splicing, acetylation, coupling to small peptides such as glutathione, and phosphorylation, this last being an important regulator of signal transduction pathways [138]. Phosphorylation is a transient and reversible metabolic process consisting on the addition of a phosphate group to a protein to either activate or deactivate its signal capability. Therefore, by measuring the phosphorylation state of proteins, it is possible to determine which signaling cascades are used in response to specific stimuli, the kinetics associated to this signaling activity, and the downstream targets that are transcribed. Furthermore, the comparison of phosphorylation activity of diseased cells to healthy samples facilitates the identification of aberrant signaling events, a useful trait to characterize diseases [139].

**Figure 9:**
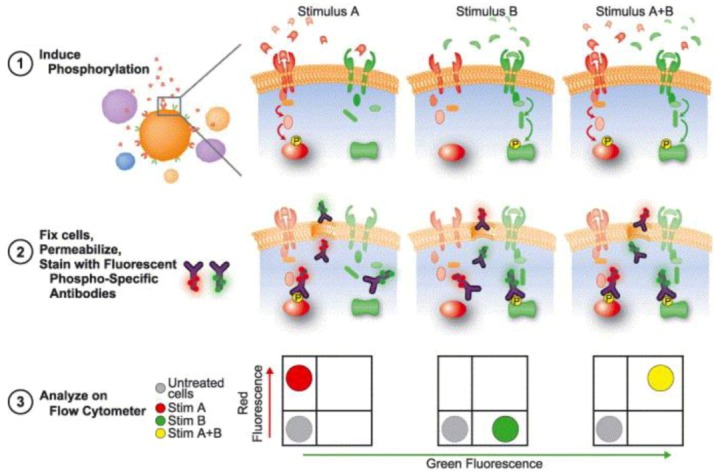
Phosphoflow process. Heterogeneous amples are first treated to induce phosphorylation with different stimuli, in this case A and B. Cells are then fixed, permeabilized and stained with fluorophore-conjugated phospho-specific antibodies, and further analyzed trough flow cytometry, where an increase in fluorescence signal reading is correlate with an increase in phosphorylation. Reprinted with permission from [141], Copyright (2004) Elsevier Ltd.

Over the last decade, a trend in flow cytometry measurements emerged to investigate the phosphorylation state of intracellular proteins, commonly called Phosphoflow and presented in Figure 9 [139]. The Phosphoflow technique allows the monitoring of effects of targeted kinase inhibitors within specific cellular populations, the detection of alteration of pathways through the use of signaling potentiators, and the monitoring of the pharmacodynamics resulting from the combination of the potentiation itself [140].

To measure these phosphorylation events, antibodies that are specific to the phosphorylated form of the protein of interest must be raised; these phosphor specific antibodies are coupled to fluorophores that can be detected and analyzed by flow cytometry, where an increase in fluorescence reading is correlated with an increase in phosphorylation [141]. Depending on the flow cytometer used, more than 13 parameters can be simultaneously analyzed in a single cell. Moreover, the multiparameter nature of flow cytometry, coupled with the possibility of both intracellular and extracellular readings, allows for subset-specific analysis to be performed in heterogeneous populations, such as whole blood samples [139]. These readings can also be performed in multi-well plates in parallel, making it a suitable option for high throughput experiments [142].

As a prime example of phosphoflow being used to understand different drug mechanisms, Shachaf and coworkers found that atorvastatin, a 3-hydroxy-3-methylglutaryl-coenzyme A (HMG-CoA) reductase inhibitor, can prevent and reverse MYC-induced lymphomagenesis by inactivation of Ras-induced ERK1/2 phosphorylation, but it could not do so in the presence of activated K-RasG12D [143]. These findings suggest that heterogeneous cancer cells could have different responses to a single drug. Hence a better treatment would require multiple drugs.

Phosphoflow has also been used to identify potential drug combinations, as is suggested by the work carried out by Galligan and coworkers [144], who analyzed peripheral blood mononuclear cells (PBMCs) from individuals diagnosed with early stage and late stage Rheumatoid Arthritis, as well as from healthy individuals and patients diagnosed with osteoarthritis. Fifteen different phosphor-epitopes were analyzed and compared between the different populations; the results gave new insights into the signaling pathways for arthritic lymphocytes: they suggest that a combined therapy of a p38 inhibitor with a STAT3 inhibitor could potentially target these cells with doses much lower than those needed for a single inhibitor therapy. 

## 4. Conclusions

Microfluidic transducers can directly sense and manipulate a single cell. Packing together an array of transducers becomes possible with the small feature size of micro devices. These two unprecedented capabilities enable us to explore the small world inside a cell and collect rich information about the cellular molecular activities.

However, information handling is a double-edged sword. On one hand, high-throughput experiments uncover critical information for identification and classification of disease and leading to targets for effective therapy. On the other hand, if we collect and analyze data without discretion we can be drowned in the ocean of data. The engineering FSC approach illustrates how we can interface with the bio-complex system and be able to efficiently treat disease with minimally processed information. Streamlining the interactions between bio-complex systems and microfluidic engineering systems through proper interfacing technology will be the next paradigm shift in advancing human health.
